# Management of Cancer-Associated Thrombosis: An Evolving Area

**DOI:** 10.3390/cancers12102999

**Published:** 2020-10-16

**Authors:** Corinne Frere, Jean M. Connors, Dominique Farge

**Affiliations:** 1 Institute of Cardiometabolism and Nutrition, INSERM UMRS_1166, GRC 27 GRECO, Sorbonne Université, F-75013 Paris, France; 2 Department of Hematology, Pitié-Salpêtrière Hospital, Assistance Publique Hôpitaux de Paris, F-75013 Paris, France; 3 Hematology Division, Brigham and Women’s Hospital, Harvard Medical School, Boston, MA 02215, USA; 4 Internal Medicine, Autoimmune and Vascular Disease Unit, Saint-Louis Hospital, Assistance Publique Hôpitaux de Paris, F-75010 Paris, France; 5 Institut Universitaire d’Hématologie, EA 3518, Université de Paris, F-75010 Paris, France; 6 Department of Medicine, McGill University, Montreal, QC H4A 3J1, Canada

**Keywords:** venous thromboembolism, cancer, low-molecular weight heparin, direct oral anticoagulant, risk assessment model

## Abstract

The management of cancer-associated thrombosis (CAT) is an evolving area. With the use of direct oral anticoagulants as a new option in the management of CAT, clinicians now face several choices for the individual cancer patient with venous thromboembolism. A personalized approach, matching the right drug to the right patient, based on drug properties, efficacy and safety, side effect profile of each drug, and patient values and preference, will probably supplant the one size fits all approach of use of only low-molecular-weight heparin in the near future. We herein present eight translational, clinical research, and review articles on recent advances in the management of CAT published in the Special Issue “Treatment for Cancer-Associated Thrombosis” of *Cancers*. For now, a multidisciplinary patient-centered approach involving a close cooperation between oncologists and other specialists is warranted to guide clinical decision making and optimize the treatment of VTE in cancer patient.

Cancer-associated thrombosis (CAT) accounts for 20% of all venous thromboembolism (VTE) events occurring in the overall population [1]. VTE remains the second cause of death in cancer patients and significantly contributes to higher morbidity and resource utilization in this patient population [2,3]. The prevalence of CAT has steadily increased during the past two decades, due to increase in cancer prevalence; the use of central venous catheters; longer survival of cancer patients due to improvements in medical anti-cancer therapies, including growth factors and hormonal therapies; advanced age of cancer patient; and better detection of incidental VTE [4].

The management of CAT is based on the use of anticoagulant drugs to treat or prevent VTE, according to standardized procedures regularly updated by clinical practices guidelines [5,6]. It can also be challenging in those patients who already undergo complex cancer treatment protocols and often have nausea and vomiting and various comorbidities such as renal failure, hepatic failure, thrombocytopenia, or brain metastases.

For many years, long-term monotherapy with low-molecular-weight heparin (LMWH) has been the standard of care for the treatment and prevention of CAT [5]. However, recent randomized controlled trials (RCTs) have demonstrated that direct oral anticoagulants (DOAC) are also effective in this setting [7,8,9,10,11,12], at the cost of an increased risk of major bleeding and clinically relevant non-major bleeding with some agents [7,8] when used for preventing recurrent VTE. With the use of DOAC as a new option in the management of CAT, clinicians now face several choices for the individual cancer patient with VTE. A personalized approach, matching the right drug to the right patient, based on drug properties, efficacy and safety, side effect profile of each drug, and patient values and preference, will supplant the one size fits all approach of use of only LMWH in the near future.

This Special Issue of *Cancers* is a collection of eight translational, clinical research, and review articles on recent advances in the management of CAT. The authors discuss the unmet needs of patients with CAT and address the limits of current therapeutic approaches by analyzing strengths and weaknesses of recent trials [13], as well as the management of incidental VTE [14,15], which account for 50% of VTE in cancer patients, and the need for individualized strategies for CAT prevention [16,17,18,19,20].

Importantly, up to 75% of VTE events occur in the outpatient setting [21] and primary thromboprophylaxis in ambulatory cancer patients has become an increasing area of interest. Although thromboprophylaxis with LMWH has been associated with approximately 50% reduction in the rates of VTE events [22], in unselected ambulatory cancer patients, the overall rate of VTE is low and prophylaxis is accompanied by a non-significant increase in the rate of major bleeding, resulting in an uncertain benefit-risk balance; therefore, VTE prophylaxis is not recommended for all cancer patients [5,6]. A patient risk stratification approach to identify patients with the highest risk of VTE, and therefore the best expected benefit from thromboprophylaxis appears to be more appropriate than using prophylaxis in all patients. The Khorana score [23], developed in 2008 and based on five readily available variables (cancer type, prechemotherapy platelet count, prechemotherapy hemoglobin level or use of red cell growth factors, prechemotherapy leucocyte count, and body mass index) is the most widely used risk assessment model (RAM) for VTE prediction in cancer patients. The Khorana score was used to stratify patients with intermediate or high-risk of VTE in the “Apixaban for the Prevention of Venous Thromboembolism in High-Risk Ambulatory Cancer Patients” (AVERT) [11] and in the “Rivaroxaban for Thromboprophylaxis in High-Risk Ambulatory Patients with Cancer” (CASSINI) [12] RCTs, which recently demonstrated the efficacy and safety of targeted VTE thromboprophylaxis with DOAC. Although the Khorana score is the best validated score currently available, in some situations such as within a single cancer type, its predictive value may not discriminate between risk categories. The main predictor of the Khorana score is the cancer type, and it has been suggested that this RAM selects cancer types with the highest risk of VTE rather than cancer patients with the highest risk of VTE [24,25].

Pancreatic cancer (PC) carry the highest risk of VTE of all malignancies. In a large prospective multicenter cohort of newly diagnosed PC patients, VTE occurred in one in five patients with a median duration of 4.49 months from PC diagnosis to VTE, highlighting the need for adequate primary thromboprophylaxis in PC patients [26]. In this Special Issue of *Cancers*, results from a meta-analysis pooling data from 1003 PC patients included in five RCTs comparing pharmacological thromboprophylaxis to placebo or no placebo control for VTE prevention demonstrated that primary thromboprophylaxis decreases the risk of VTE by 69%, without increase in the risk of major bleeding [16]. This systematic review and meta-analysis included data from a subgroup of PC patients enrolled in a DOAC placebo-controlled trial for primary VTE prevention [27]. The results support the most recent ITAC [5] and ASCO [6] guidelines that recommend primary prophylaxis using either LMWH or DOAC for all ambulatory PC patient receiving systemic anticancer therapy and with a low risk of bleeding.

The net clinical benefit of primary thromboprophylaxis in other cancer types, such as primary brain tumors, hematological malignancies, or lung cancers, has not yet been firmly established. Given the inability of current RAMs to reliably discriminate between patients at high risk and those at low- or intermediate-risk for VTE in a specific cancer type [25], there is still a need to improve VTE prediction by integrating new variables into RAMs.

In their narrative review, Muster et al. summarized current knowledge on the incidence, risk factors, and challenges in the management of CAT in patients with glioblastoma [17]. They highlighted the major efforts currently underway to identify accurate and reliable biomarkers to predict the risk of VTE in these patients. Mir Seyed Nazari et al. conducted an original study to assess the impact of nine anti- and pro-inflammatory cytokines on the risk of thromboembolic events in 76 patients with glioma prospectively enrolled in the observational Vienna Cancer and Thrombosis Study [18]. They reported an inverse association between chemokine C-C motif ligand 3 (CCL3) levels and the risk of VTE (hazard ratio per double increase, 0.385; 95% confidence interval (95% CI), 0.161–0.925; *p* = 0.033), indicating that CCL3 might be used as a biomarker to identify glioma patients at higher-risk of VTE who may benefit from thromboprophylaxis [18]. Likewise, Oto et al. investigated the association among the expression of 179 microRNAs (miRNAs), neutrophil activation markers (including cell-free DNA, nucleosomes, calprotectin, and myeloperoxidase), and the risk of VTE in a cohort of 100 patients with primary brain tumors undergoing surgery. In their study, a model incorporating myeloperoxidase and miR-140-3p expression levels predicted post-surgical pulmonary embolism (PE) in glioma patients with both high sensitivity and high specificity (area under the Receiver Operator Curve = 0.79; 95% CI, 0.64–0.9]) [19], an improvement over available risk prediction scores. Together, these studies illustrate how the identification of new predictive biomarkers might help to refine VTE risk prediction. However, the absence of external validation and use of these specific biomarkers that are not readily available in clinical laboratories still preclude their use in daily practice.

Incorporation of genomic data into RAMs also represents an important step forward to improve VTE risk prediction. Some germline single-nucleotide polymorphisms (SNPS) known to influence the risk of VTE in the general population, such as rs6025 and rs4524 in the *F5* gene, rs5985 in the *F13* gene and rs2232698 in the *SERPINA10* gene, have been demonstrated to be significant predictors for VTE in cancer patients [28]. The incorporation of these four SNPs into the TiC-Onco risk score showed promising results in patients with solid cancers, although these results require external validation [28]. However, studies aiming to identify novel VTE-susceptibility SNPs in cancer patients remain scarce. Mateos et al. should be congratulated on conducting two separate genome-wide association studies (GWAS) in two independent large cohorts (NOPHO and ERASE) of children treated for acute lymphoblastic leukemia (ALL) [20]. They genotyped 10,922,653 common germline SNPs using an Illumina HumanExome BeadChip and subsequently performed a GWAS meta-analysis. Two top-SNPs, rs1804772 in the *ALOX15B* gene and rs570684 in the *KALRN* gene, were identified as potential SNPs of interest to stratify ALL children with high risk of VTE who may benefit from thromboprophylaxis. Larger sample size studies are needed to confirm these findings. In addition, tumors specific mutations have been recently associated with the risk of VTE in some cancers, including non-small cell lung carcinoma, colon cancer, and myeloproliferative neoplasms, and integration of tumors mutational signature into VTE RAMs may also significantly improve VTE risk prediction in the near future [29,30].

The management of VTE in cancer patients continues to be an evolving area. Whether complex models based on machine learning-driven approaches will allow us to significantly refine VTE risk prediction and improve appropriate use of VTE prophylaxis in the future remains an unanswered question. For now, a multidisciplinary patient-centered approach involving a close cooperation between oncologists and other specialists is warranted to guide clinical decision making and optimize the treatment of VTE in cancer patient.
